# Model Parameter Adaption-Based Multi-Model Algorithm for Extended Object Tracking Using a Random Matrix

**DOI:** 10.3390/s140407505

**Published:** 2014-04-24

**Authors:** Borui Li, Chundi Mu, Shuli Han, Tianming Bai

**Affiliations:** 1 Department of Automation, Tsinghua University, Beijing 100084, China; E-Mail: muchd@mail.tsinghua.edu.cn; 2 Navy Armament Academy, Beijing 100036, China; E-Mail: btm_zn@126.com; 3 National Meteorological Information Center, Beijing 100081, China; E-Mail: hansl@cma.gov.cn

**Keywords:** extended object tracking, Bayesian approach, random matrix, interacting multi-model algorithm, model parameter adaption

## Abstract

Traditional object tracking technology usually regards the target as a point source object. However, this approximation is no longer appropriate for tracking extended objects such as large targets and closely spaced group objects. Bayesian extended object tracking (EOT) using a random symmetrical positive definite (SPD) matrix is a very effective method to jointly estimate the kinematic state and physical extension of the target. The key issue in the application of this random matrix-based EOT approach is to model the physical extension and measurement noise accurately. Model parameter adaptive approaches for both extension dynamic and measurement noise are proposed in this study based on the properties of the SPD matrix to improve the performance of extension estimation. An interacting multi-model algorithm based on model parameter adaptive filter using random matrix is also presented. Simulation results demonstrate the effectiveness of the proposed adaptive approaches and multi-model algorithm. The estimation performance of physical extension is better than the other algorithms, especially when the target maneuvers. The kinematic state estimation error is lower than the others as well.

## 1. Introduction

During the past several decades, the resolution of radar and other sensors continues to increase, thereby generating complex requirements for object tracking (OT) technology [1]. Traditional OT technology characterizes the target as a point source object whose physical extension is ignored [2]. The centroid is usually utilized to represent the target, and its kinematic state such as position, velocity, and acceleration is estimated in OT. This simplification is meaningful only when physical extension can be neglected compared with the sensors' measurement error. However, considering the increasing sensor resolution, physical extension state should be estimated jointly in OT approaches, especially when tracking relatively large targets such as aircraft carriers and large transport planes. This method is referred to as extended object tracking (EOT) [1,3–5]. When targets form a closely spaced formation (e.g., an aircraft formation), the individual target becomes indistinguishable from the formation because of limited sensor capabilities and sensor-to-target geometry. Thus, closely spaced group objects can also be considered extended objects [1,3]. Data association in EOT is difficult because extended objects produce a highly varying number of measurements corresponding to a single extended object [3].

A number of techniques and approaches have been proposed to solve the EOT problem, which requires estimating physical extension and the centroid's kinematic state jointly. Baum *et al.* [6] proposed a novel modeling approach to describe physical extension. The proposed model was called random hypersurface model. A recursive **Bayesian** estimator was derived based on this model. Mahler [7,8] applied probability hypothesis density (PHD) filter and cardinalized PHD (CPHD) filter to EOT, including both extended and unresolved targets. Vo *et al.* [9] presented a mathematically strict Bayesian filter for EOT, which could be reduced to a CPHD filter under some assumptions when tracking a single target. Carmi *et al.* [10] utilized the Gaussian mixture model to describe the time-varying dynamics of group objects. A Markov chain Monte Carlo particle filter for multi-target tracking was then obtained. Multiple hypotheses tracking was also a feasible option for EOT [11]. Zhang and Bar-Shalom [12] proposed a new framework based on multiple kernel centers for visual object tracking.

**Figure 1.**
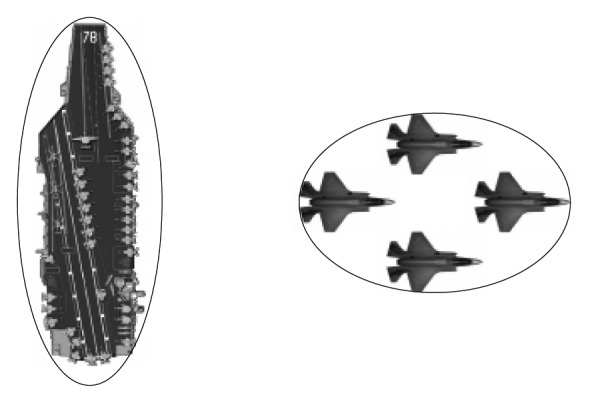
Description by ellipses of the target's physical extension. A large ship (**left**) and an aircraft formation (**right**).

Koch [1] proposed the random matrix-based approach for extended object tracking. A recursive form filter is derived within the Bayesian framework, and the final form of this random matrix-based filter (RMF) is more or less similar to that of the standard Kalman filter. RMF describes the physical extension of the target with an ellipse or ellipsoid. Thus, RMF is reasonable and can be applied in many practical applications as shown in Figure 1. Given that symmetrical positive definite (SPD) matrices can represent all the ellipses/ellipsoids centering at the origin, RMF utilizes the random SPD matrix to characterize the physical extension of the target. The random SPD matrix is assumed to obey Wishart-related distributions. Then the Bayesian formula can be applied to obtain a very simple filter according to the properties of the Kronecker product and Gaussian and Wishart-related distributions. Feldmann [3] introduced a more accurate distribution of measurement noise with respect to the target's physical extension and derived a new RMF approach. This new approach can be integrated into an interacting multi-model (IMM) framework to improve estimation performance if the object state switches between maneuvering and non-maneuvering. However, the derivation process cannot be maintained within the Bayesian framework, and several approximations are applied to obtain a recursive form filter. Lan and Li [4,5] further improved extension dynamic model and measurement noise model by describing extension evolution and observation distortion with real matrices ***A***_k_ and ***B***_k_. This improvement is logical given the relationship between the SPD matrix and ellipse/ellipsoid. It can be applied within the Bayesian framework, which requires only an insignificant approximation.

RMF approach is much simpler than the other EOT approaches mentioned above, thereby making it more promising. The simulation results demonstrate the effectiveness of the three abovementioned RMF approaches. However, the physical extension estimation error obtained by these approaches significantly increases when the target maneuvers (e.g., turning motion). The extension dynamic model in [4] is improved in this study based on RMF approach to simplify the description of extension evolution. Model parameter adaptive approaches for both extension dynamic and measurement noise are proposed based on properties of SPD matrix and ellipse/ellipsoid. The proposed adaptive RMF is called ARMF. The preliminary results of single-model ARMF have been published in a previous conference paper [13]. A multi-model algorithm is proposed by integrating ARMF into the widely utilized IMM framework to further improve the performance of RMF when the target maneuvers. Simulation results show that the ARMF approach and ARMF-based multi-model (ARMF-MM) algorithm are effective.

## 2. Bayesian EOT Using Random Matrix

The dynamic state of an extended object at scan *k* is described by both the centroid's kinematic state ***x***_k_ and physical extension ***X***_k_, where ***x***_k_ is an *sd* × 1 random vector and ***X***_k_ is a *d* × *d* SPD random matrix. Spatial dimension *d* is usually set to 2 or 3 in EOT. *s* is the dimension of the state in one spatial dimension. *s* = 2 means that position and velocity are considered. If acceleration is also considered, then *s* = 3.

The basic idea of random matrix-based EOT is to characterize joint density *p*[*x*_k_,***X***_k_|***Z***^k^] as a product of a Gaussian density and an inverted Wishart density based on Bayes' formula:

(1) $$\begin{array}{c} p[{\text{x}}_{k},{\text{X}}_{k}|{\text{Z}}^{k}]=\frac{p[{\text{Z}}_{k}|x_{k},{\text{X}}_{k}]\times p[{\text{x}}_{k},{\text{X}}_{k}|{\text{Z}}^{k-1}]}{\iint p[{\text{Z}}_{k}|{\text{x}}_{k},{\text{X}}_{k}]\times p[{\text{x}}_{k},{\text{X}}_{k}|{\text{Z}}^{k-1}]\text{d}x_{k}\text{d}{\text{X}}_{k}}\\ =\frac{1}{c}p[{\text{Z}}_{k}|{\text{x}}_{k},{\text{X}}_{k}]\times p[{\text{x}}_{k},{\text{X}}_{k}|{\text{Z}}^{k-1}] \end{array}$$

where ${\text{Z}}_{k}≜{\left\{{\text{z}}_{k}^{r}\right\}}_{r=1}^{n_{k}}$ denotes the set of *n_k_* measurements at scan *k*, *n_k_* ≥ 1 is assumed to be independent of ***x***_*k*_ and ***X***_*k*_, ${\text{Z}|}^{k}≜{\left\{{\text{z}}_{j}\right\}}_{j=1}^{k}$, and *c* always denotes the normalization factor in this paper.

This section summarizes the key results and steps. More detailed assumptions and derivations can be found in [1,3,4,14].

### 2.1. Dynamic and Measurement Models

The dynamic model of ***x***_*k*_ for EOT is very similar to the models utilized in many Kalman-related filters:

(2) $${\text{x}}_{k}={\text{F}}_{k}{\text{x}}_{k-1}+{\text{ω}}_{k}$$

where ${\text{F}}_{k}={\tilde{\text{F}}}_{k}⊗{\text{I}}_{d}$, “⊗” is the right Kronecker product [15], ***F̃****_k_* represents the state matrix in one spatial dimension, and ***I***_*d*_ is the *d* × *d* identity matrix. Independent process noise ***ω***_k_ follows a normal distribution [1]:

(3) $${\text{ω}}_{k}∼N(0,{\tilde{\text{D}}}_{k}⊗{\text{I}}_{d})=N(0,{\text{Q}}_{k})$$

where N(***μ***,***Σ***) stands for normal distribution with mean ***μ*** and covariance matrix ***Σ***, and ***D̃****_k_* denotes the covariance matrix of ***ω***_k_ in one spatial dimension. ***F̃****_k_* and ***D̃****_k_* can be selected according to specific conditions of EOT. Singer's model [1], constant velocity model, and constant acceleration model [16] are all feasible alternatives.

The following assumptions are introduced for physical extension [1,4]:

(4) $$p[{\text{X}}_{k}|{\text{X}}_{k-1}]=W({\text{X}}_{k};\delta_{k},{\text{A}}_{k}{\text{X}}_{k-1}{\text{A}}_{k}^{T}/\delta_{k})$$

(5) $$p[{\text{X}}_{k-1}|{\text{Z}}^{k-1}]=ℐW({\text{X}}_{k-1};{\text{v}}_{k-1|k-1},{\text{X}}_{k-1|k-1})$$

where *δ*_k_ > *d* − 1 stands for the degrees of freedom, *ν*_k−1|k−1_ > 2*d* is a scalar parameter, subscript *k*−1|*k*−1 indicates the estimated value of corresponding variable at scan *k* − 1, and ***A***_*k*_ is a *d* × *d* nonsingular real matrix describing physical extension evolution.

W(***X***; *a*,***A***), the Wishart density of SPD matrix ***X***, is defined by:

(6) $$W(\text{X};a,\text{A})=\frac{1}{c}{\left|\text{A}\right|}^{-(1/2)a}{\left|\text{X}\right|}^{\left(1/2)(a-d-1\right)}\text{etr}[-\frac{1}{2}{\text{A}}^{-1}\text{X}]$$

where etr[·] is short for exp[trace(·)], *a* ≥ *d*, and expectation E[***X***] = *a****A***.

IW(***X***; *a*,***A***), the inverted Wishart density of SPD matrix ***X***, is defined by:

(7) $$ℐW(\text{X};a,\text{A})=\frac{1}{c}{\left|\text{A}\right|}^{\left(1/2)(a-d-1\right)}{\left|\text{X}\right|}^{(-1/2)a}\text{etr}[-\frac{1}{2}\text{A}{\text{X}}^{-1}]$$

with *a* > 2*d* + 2 and expectation E[***X***] = ***A***/(*a* − 2*d* − 2).

The assumption in Equation (4) is different from that in Equation (10) in [4]. It is simpler and more plausible, which will be explained in Section 3.1.

Measurement $z_{k}^{r}$ is modeled as follows:

(8) $${\text{z}}_{k}^{r}={\text{H}}_{k}{\text{x}}_{k}+{\text{υ}}_{k}^{r}$$

where ${\text{H}}_{k}={\tilde{\text{H}}}_{k}⊗{\text{I}}_{d}$ and ${\tilde{\text{H}}}_{k}=[1,0,\cdots ,0]$ denotes the meas–urement matrix in one spatial dimension [1].

Gaussian measurement noise ${\text{ν}}_{k}^{r}$ is independent of ${\text{ν}}_{k}^{j}$ (*j* = 1,2,…,*n_k_*, *j ≠ r*). Its distribution is assumed to be [4]:

(9) $${\text{υ}}_{k}^{r}∼N(0,{\text{B}}_{k}{\text{X}}_{k}{\text{B}}_{k}^{\text{T}})$$

where ***B***_k_ is a *d* × *d* nonsingular real matrix describing the measurement distortion of extension. Equation (9) indicates that the measurements are affected by physical extension ***X***_k_.

### 2.2. Prediction

The prediction density of ***x***_k_ and ***X***_k_ can be factorized as:

(10) $$p[{\text{x}}_{k},{\text{X}}_{k}|{\text{Z}}^{k-1}]=p[{\text{x}}_{k}|{\text{X}}_{k},{\text{Z}}^{k-1}]\times p[{\text{X}}_{k}|{\text{Z}}^{k-1}]$$

The first factor on the right of the previous equation is assumed to have the following structure [1]:

(11) $$\begin{array}{l} p[{\text{x}}_{k}|{\text{X}}_{k},{\text{Z}}^{k-1}]=\int p[{\text{x}}_{k}|{\text{X}}_{k},{\text{x}}_{k-1},{\text{Z}}^{k-1}]\times p[{\text{x}}_{k-1}|{\text{X}}_{k},{\text{Z}}^{k-1}]\text{d}{\text{x}}_{k-1}\\ =\int N({\text{x}}_{k};{\text{F}}_{k}{\text{x}}_{k-1},{\text{Q}}_{k})\times N({\text{x}}_{k-1};{\text{x}}_{k-1|k-1},{\text{P}}_{k-1|k-1})\text{d}{\text{x}}_{k-1}\\ =N({\text{x}}_{k};{\text{x}}_{k|k-1},{\text{P}}_{k|k-1}) \end{array}$$

where subscript *k*|*k*−1 indicates the predicted value of corresponding variable at scan *k*.

We then obtain the prediction equations of kinematic state:

(12) $$\begin{array}{c} {\text{x}}_{k|k-1}={\text{F}}_{k}{\text{x}}_{k-1|k-1}\\ {\text{P}}_{k|k-1}={\text{F}}_{k}{\text{P}}_{k-1|k-1}{\text{F}}_{k}^{T}+{\text{Q}}_{k} \end{array}$$

*p*[***X***_k_|***Z***^k−1^] can be calculated based on Equations (4) and (5):

(13) $$\begin{array}{c} p[{\text{X}}_{k}|{\text{Z}}^{k-1}]=\int p[{\text{X}}_{k}|{\text{X}}_{k-1}]\times p[{\text{X}}_{k-1}|{\text{Z}}^{k-1}]\text{d}{\text{X}}_{k-1}\\ =\int W({\text{X}}_{k};\delta_{k},{\text{A}}_{k}{\text{X}}_{k-1}{\text{A}}_{k}^{\text{T}}/\delta_{k})\times IW({\text{X}}_{k-1};{\text{v}}_{k-1|k-1},{\text{X}}_{k-1|k-1})\text{d}{\text{X}}_{k-1}\\ =GB_{d}^{II}(X_{k};\delta_{k}/2,({\text{v}}_{k-1|k-1}-d-1)/2,{\text{A}}_{k}{\text{X}}_{k-1|k-1}{\text{A}}_{k}^{T}/\delta_{k},0) \end{array}$$

where $GB_{\text{d}}^{\text{II}}\left(\cdot \right)$ is the probability density function (pdf) of “generalized beta type II” distribution. An inverted Wishart distribution is utilized to obtain a recursive-form estimator and approximate $GB_{d}^{II}\left(\cdot \right)$ via first and second moment matching [1,4]:

(14) $$p[{\text{X}}_{k}|{\text{Z}}^{k-1}]\approx IW({\text{X}}_{k};{\text{v}}_{k|k-1},{\text{X}}_{k|k-1})$$

where:

(15) $$v_{k|k-1}=\frac{\delta_{k}(\lambda_{k-1}+1)(\lambda_{k-1}-1)(\lambda_{k-1}-2)}{\lambda_{k-1}^{2}(\delta_{k}+\lambda_{k-1})}+2d+4$$

and:

(16) $${\text{X}}_{k|k-1}=({\text{v}}_{k|k-1}-2d-2){\text{A}}_{k}{\text{X}}_{k-1|k-1}{\text{A}}_{k}^{T}/\lambda_{k-1}$$

with *λ*_k−1_ = *ν*_k−1|k−1_ − 2*d* − 2.

The substitution of Equations (11) and (14) into Equation (10) yields:

(17) $$p[{\text{x}}_{k},{\text{X}}_{k}|{\text{Z}}^{k-1}]=N({\text{x}}_{k};{\text{x}}_{k|k-1},{\text{P}}_{k|k-1})\times IW({\text{X}}_{k};{\text{v}}_{k|k-1},{\text{X}}_{k|k-1})$$

### 2.3. Update

In consideration of Equation (1) and based on Equations (8) and (9), likelihood function *p*[***Z***_k_|*x*_k_,***X***_k_] is obtained as follows [4]:

(18) $$\begin{array}{c} p[{\text{Z}}_{k}|{\text{x}}_{k},{\text{X}}_{k}]\propto p[{\text{Z}}_{k}|n_{k},{\text{x}}_{k},{\text{X}}_{k}]=\overset{n_{k}}{\underset{r=1}{\text{∏}}}N({\text{z}}_{k}^{r};{\text{H}}_{k}{\text{x}}_{k},{\text{B}}_{k}X_{k}{\text{B}}_{k}^{\text{T}})\\ \propto N({\bar{\text{z}}}_{k};{\text{H}}_{k}{\text{x}}_{k},\frac{{\text{B}}_{k}{\text{X}}_{k}{\text{B}}_{k}^{T}}{n_{k}})\text{W}({\bar{\text{Z}}}_{k};n_{k}-1,{\text{B}}_{k}{\text{X}}_{k}{\text{B}}_{k}^{T}) \end{array}$$

where:

(19) $${\bar{\text{z}}}_{k}=\frac{1}{n_{k}}\sum_{r=1}^{n_{k}}{\text{z}}_{k}^{r},\;{\bar{\text{Z}}}_{k}=\sum_{r=1}^{n_{k}}({\text{z}}_{k}^{r}-{\bar{\text{z}}}_{k}){\left({\text{z}}_{k}^{r}-{\bar{\text{z}}}_{k}\right)}^{\text{T}}$$

The substitution of Equations (17) and (18) into Equation (1) yields [1,4]:

(20) $$\begin{array}{c} \begin{array}{c} p[{\text{x}}_{k},{\text{X}}_{k}|{\text{Z}}^{k}]\propto N({\bar{\text{z}}}_{k};{\text{H}}_{k}{\text{x}}_{k},\frac{{\text{B}}_{k}{\text{X}}_{k}{\text{B}}_{k}^{T}}{n_{k}})W({\bar{\text{Z}}}_{k};n_{k}-1,{\text{B}}_{k}{\text{X}}_{k}{\text{B}}_{k}^{T})\\ \times N({\text{x}}_{k};{\text{x}}_{k|k-1},{\text{P}}_{k|k-1})\times \text{IW}({\text{X}}_{k};{\text{v}}_{k|k-1},{\text{X}}_{k|k-1}) \end{array} \end{array}$$

The product of the two Gaussian probability density functions in the previous equation can be converted to [1,4]:

(21) $$\text{N}({\text{x}}_{k};{\text{x}}_{k|k-1},{\text{P}}_{k|k-1})\times \text{N}({\bar{\text{z}}}_{k};{\text{H}}_{k}{\text{x}}_{k},\frac{{\text{B}}_{k}{\text{X}}_{k}{\text{B}}_{k}^{T}}{n_{k}})=\text{N}({\bar{\text{z}}}_{k};{\text{H}}_{k}{\text{x}}_{k|k-1},{\text{S}}_{k})\times \text{N}({\text{x}}_{k};{\text{x}}_{k|k},{\text{P}}_{k|k})$$

with:

(22) $$\begin{array}{c} {\text{x}}_{k|k}={\text{x}}_{k|k-1}+{\text{K}}_{k}({\bar{\text{z}}}_{k}-{\text{H}}_{k}{\text{x}}_{k|k-1})\\ {\text{P}}_{k|k}={\text{P}}_{k|k-1}-{\text{K}}_{k}{\text{S}}_{k|k-1}{\text{K}}_{k}^{\text{T}} \end{array}$$

and:

(23) $$\begin{array}{c} {\text{S}}_{k|k-1}={\text{H}}_{k}{\text{P}}_{k|k-1}{\text{H}}_{k}^{\text{T}}+\frac{{\text{B}}_{k}{\text{X}}_{k}{\text{B}}_{k}^{T}}{n_{k}}\approx {\text{H}}_{k}{\text{P}}_{k|k-1}{\text{H}}_{k}^{T}+\frac{{\text{B}}_{k}{\text{X}}_{k|k-1}{\text{B}}_{k}^{T}}{n_{k}}\\ {\text{K}}_{k}={\text{P}}_{k|k-1}{\text{H}}_{k}^{T}{\text{S}}_{k|k-1}^{-1} \end{array}$$

where ***X***_*k*|*k*−1_ is used to approximate ***X***_*k*_.

Based on Equations (21) and (20) can be rewritten as [1,4]:

(24) $$\begin{array}{l} \begin{array}{c} p[{\text{x}}_{k},{\text{X}}_{k}|{\text{Z}}^{k}]\propto \text{W}({\bar{\text{Z}}}_{k};n_{k}-1,{\text{B}}_{k}{\text{X}}_{k}{\text{B}}_{k}^{\text{T}})\text{IW}({\text{X}}_{k};{\text{v}}_{k|k-1},{\text{X}}_{k|k-1})\\ \times \text{N}({\bar{\text{z}}}_{k};{\text{H}}_{k}{\text{x}}_{k|k-1},{\text{S}}_{k|k-1})\text{N}({\text{x}}_{k};{\text{x}}_{k|k},{\text{P}}_{k|k}) \end{array} \end{array}$$

Subsequently, the first three pdfs on the right side of the previous equation can be combined as IW(***X***_k_; ***ν***_k|k_,***X***_k|k_) with:

(25) $$\begin{array}{l} {\text{X}}_{k|k}={\text{X}}_{k|k-1}+{\text{B}}_{k}^{-1}{\bar{\text{Z}}}_{k}{\text{B}}_{k}^{-\text{T}}+{\text{N}}_{k|k-1}\\ {\text{N}}_{k|k-1}=({\bar{\text{z}}}_{k}-{\text{H}}_{k}x_{k|k-1}){\left({\bar{\text{z}}}_{k}-{\text{H}}_{k}{\text{x}}_{k|k-1}\right)}^{\text{T}}{\text{S}}_{k|k-1}^{-1}{\text{X}}_{k}\\ \approx ({\bar{\text{z}}}_{k}-{\text{H}}_{k}{\text{x}}_{k|k-1}){\left({\bar{\text{z}}}_{k}-{\text{H}}_{k}{\text{x}}_{k|k-1}\right)}^{\text{T}}{\text{S}}_{k|k-1}^{-1}{\text{X}}_{k|k-1}\\ \approx {\text{X}}_{k|k-1}^{1/2}{\text{S}}_{k|k-1}^{-1/2}({\bar{\text{z}}}_{k}-{\text{H}}_{k}{\text{x}}_{k|k-1}){\left({\bar{\text{z}}}_{k}-{\text{H}}_{k}{\text{x}}_{k|k-1}\right)}^{\text{T}}{\left({\text{S}}_{k|k-1}^{-1/2}\right)}^{\text{T}}{\left({\text{X}}_{k|k-1}^{1/2}\right)}^{\text{T}}\\ v_{k|k}=v_{k|k-1}+n_{k}-1 \end{array}$$

where ${\text{X}}_{k|k-1}^{1/2}$ and ${\text{S}}_{k|k-1}^{-1/2}$ are square roots of SPD matrices ***X***_*k*|*k*−1_ and ${\text{S}}_{k|k-1}^{-1}$, which can be obtained by Cholesky factorization. They are utilized to keep ***N***_*k*|*k*−1_ and ***X***_*k*|*k*_ in positive definite or positive semi-definite structure [3]. The derivation of Equation (25) is similar to the corresponding process in [1].

Then, *p*[*x*_*k*_,***X***_*k*_|***Z***^*k*^] is converted to the product of a Gaussian density and an inverted Wishart density as follows:

(26) $$p[{\text{x}}_{k},{\text{X}}_{k}|{\text{Z}}^{k}]\propto \text{N}({\text{x}}_{k};{\text{x}}_{k|k},{\text{P}}_{k|k})\text{IW}({\text{X}}_{k};{\text{v}}_{k|k},{\text{X}}_{k|k})$$

The expectation of physical extension ***X***_*k*_ is employed as the final extension estimation:

(27) $${\bar{\text{X}}}_{k}=\text{E}[{\text{X}}_{k}|{\text{Z}}^{k}]={\text{X}}_{k|k}/\alpha_{k|k}$$

where *α*_*k*|*k*_ = *ν*_*k*|*k*_ − 2*d* – 2 > 0.

The Bayesian recursive estimator for EOT with random matrix (*i.e.*, RMF) is composed of Equations (12), (15), (16), (22), (23), (25) and (27). A notable similarity exists between the standard Kalman filter and the centroid's kinematic part of RMF.

## 3. Model Parameter Adaptive Approaches

For a certain model used in multi-model (MM) algorithm, the model parameters include ***D̃****_k_* in Equation (3), *δ*_k_ and ***A***_k_ in Equation (4)***B***_k_ in Equation (9), *etc.* The model parameter adaptive approaches act on extension dynamic model parameter ***A***_k_ and measurement noise model parameter ***B***_k_ based on the relationship between the SPD matrix and ellipse/ellipsoid. The basic principle of parameter adaptive approach when *d* = 2 and *d* = 3 is the same. *d* = 2 is regarded as an example to introduce the parameter adaptive approaches.

The following assignments are adopted in the sequel for the purpose of convenience: *d* = 2, *s* = 3, and ${\tilde{\text{H}}}_{k}=[1,0,0]$, which means that the kinematic state in one spatial dimension is [position, velocity, acceleration]^T^ and only the position coordinates of the target's centroid are measured. Therefore, the ellipse describing physical extension can be represented by [4]:

(28) $${\left(\vec{\text{x}}-{\text{H}}_{k}{\text{x}}_{k}\right)}^{T}{\text{X}}_{k}^{-1}(\vec{\text{x}}-{\text{H}}_{k}{\text{x}}_{k})=1$$

with ***x̄*** denoting the coordinates of the points on the ellipse.

The following factors must be identified when determining an ellipse on the 2D plane.

(1) Location: the coordinates of the ellipse's center point, which are determined by kinematic state ***x****_k_*;
(2) Size: the lengths of the semi-axes, which are equal to the positive square roots of ***X****_k_*'s eigenvalues;
(3) Orientation: the direction of the semi-axes, which is determined by the angle between either of the semi-axes and either of the coordinate axes when *d* = 2. Several definitions of orientation angles are available. For example, the angle between the major semi-axis and x axis is suitable when the counterclockwise direction is considered the positive direction.

Size and orientation are determined entirely by physical extension ***X_k_***. As long as the size or orientation of an ellipse changes, the SPD matrix representing the ellipse would change. This occurrence means that the SPD matrices represent only the ellipses centering at the origin. However, all the ellipses centering at any position on the 2D plane will be covered if ***X_k_*** is combined with kinematic state ***x_k_***.

The following lemma is necessary to decompose SPD matrix ***X_k_*** into a form wherein the relationship between ***X_k_*** and the ellipse can be easily analyzed.

**Lemma 1.** *If **X** is a d* × *d SPD matrix, then **X** can be transformed to a diagonal matrix by up to* $\frac{d(d-1)}{2}$ *“Givens rotations” [17]. When d* = *2, **X** can be transformed as follows:*

(29) $$R^{T}(\varphi^{\text{G}})XR(\varphi^{\text{G}})=\text{diag}(\sigma_{1}^{*},\sigma_{2}^{*})$$

*where* $R(\varphi^{\text{G}})=\left[\begin{array}{cc} \cos\varphi^{\text{G}} & -\sin\varphi^{\text{G}}\\ \sin\varphi^{\text{G}} & \cos\varphi^{\text{G}} \end{array}\right]$ *is the rotation matrix,* $\sigma_{1}^{*}$ *and* $\sigma_{2}^{*}$ *are the eigenvalues of **X***, $\varphi^{\text{G}}\in [-\frac{\pi }{2},\frac{\pi }{2})$*, and*

(30) $$\varphi^{\text{G}}=\text{arctan}\left(-\rho \pm \sqrt{1+\rho^{2}}\right)$$

*with* $\rho =\frac{X(1,1)-X(2,2)}{2X(1,2)}$.

*Therefore,*

(31) $$X=R(\varphi^{\text{G}})\text{diag}(\sigma_{1}^{*},\sigma_{2}^{*})R^{T}(\varphi^{\text{G}})$$

**Proof of Lemma 1.** Expand the left portion of Equation (29), then the following equation is obtained by setting the non-diagonal elements to 0:

(32) $$X(1,2)\tan^{2}(\varphi^{\text{G}})+(X(1,1)-X(2,2))\tan(\varphi^{\text{G}})-X(1,2)=0$$

Let *ρ* denote $\frac{X(1,1)-X(2,2)}{2X(1,2)}$; thus:

(33) $$\tan(\varphi^{\text{G}}(X))=-\rho \pm \sqrt{1+\rho^{2}}$$

*Q.E.D*.

Decomposing SPD matrix ***X*** into the product of orthogonal matrices and a diagonal matrix is relatively easy in mathematics. Except for “Givens rotations,” singular value and eigenvalue decomposition are also possible. However, the orthogonal matrix contains not only rotation transformation but also symmetry transformation (rotation matrix is the orthogonal matrix whose determinant is +1). The form and sequence of symmetry transformation are uncertain. Thus, neither of the two decomposition methods is applicable for model parameter adaption.

According to Lemma 1, SPD matrix ***X_k_*** representing a 2D ellipse could be factorized as follows:

(34) $$\begin{array}{ll} X_{k} & =R(\varphi_{k})\text{diag}(\sigma_{k,1},\sigma_{k,2})R^{T}(\varphi_{k})\\ & ≜R(\varphi_{k})V_{k}R^{T}(\varphi_{k}) \end{array}$$

where *σ_k_*_,1_ and *σ_k_*_,2_ are ***X_k_***'s eigenvalues describing the ellipse's size, and $\varphi_{k}\in \left[-\frac{\pi }{2},\frac{\pi }{2}\right)$ is the orientation angle describing the ellipse's orientation.

### 3.1. Extension Dynamic Model Parameter Adaption

From the extension dynamic model in Equation (4), we obtain:

(35) $$\text{E}[X_{k}|X_{k-1}]=A_{k}X_{k-1}A_{k}^{T}$$

No *δ_k_* exists on the right side of Equation (35), which is different from Equation (11) in [4]. However, employing Equation (35) allows ***A_k_*** to cover all the possible changes in the size and orientation of the ellipses represented by SPD matrices ***X_k_*** and ***X_k_***_-1_. According to Equation (15), *δ_k_* is still incorporated in the Bayesian RMF estimator, describing the uncertainty of physical extension evolution [1]. Therefore, the improvement of Equation (4) is reasonable and meaningful.

For parameter adaption when *d* = 2, we propose to calculate ***A_k_*** by the following equation:

(36) $$A_{k}=R(\theta_{k}^{A})\text{diag}(\gamma_{k}^{A,1},\gamma_{k}^{A,2})$$

where $\theta_{k}^{A}\in \left[-\frac{\pi }{2},\frac{\pi }{2}\right)$ describes the change in ellipse's orientation and $\gamma_{k}^{A,1}\geq \gamma_{k}^{A,2}>0$ are scalars describing the change in size.

Although the range of ***A_k_*** is reduced, ***A_k_*** defined by Equation (36) is still able to approximately describe all changes in the ellipses' size and orientation.

According to Equations (35) and (36), $\theta_{k}^{A}$ describes the change amount of orientation angles of the two ellipses represented by ***X_k_*** and ***X***_k−1_ respectively. Then $\theta_{k}^{A}$ could be set to Δ*φ_k_* = *φ_k_* − *φ_k_*_−1_, where *φ_k_* and *φ_k_*_−1_ are orientation angles described in Equation (34). Let *φ^G^*(***X_k_***) and *φ^G^*(***X_k_***_−1_) denote the angles calculated by Lemma 1. If the difference between *φ^G^*(**·**) and *φ_k_* is a constant (*i.e.*, *φ^G^*(***X_k_***) − *φ^G^*(***X_k_***_−1_) = Δ*φ_k_*), then $\theta_{k}^{A}$ could be set to *φ^G^*(***X_k_***) − *φ^G^*(***X***_k−1_). However, no matter “+” or “−” in Equation (30) is selected, the difference between *φ^G^*(**·**) and *φ_k_* is not a constant, *i.e.*, *φ^G^*(***X_k_***) − *φ^G^*(***X***_k−1_) ≠ Δ*φ_k_*. Thus, the following two restrictions are introduced.

First of all, at the very beginning of EOT, the initial value of extension state should be selected by:

(37) $${\bar{X}}_{0}=R(\varphi_{0})\text{diag}(\sigma_{0,1},\sigma_{0,2})R^{T}(\varphi_{0})$$

According to the previous discussion and Equation (34), ***X̅***_0_ should be able to cover all the possible SPD matrices representing the ellipses centering at the origin.

Secondly, we select “+” or “−” in Equation (30) to satisfy the following condition:

(38) $$\text{sign}(\sigma_{1}-\sigma_{2})=\text{sign}(\sigma_{0,1}-\sigma_{0,2})$$

where *σ*_1_ and *σ*_2_ are the same as those in Equation (29), and sign(·) is the sign function.

If these two restrictions are met, then *φ*^*G*^(**·**) differs from *φ_k_* by a constant during the whole EOT process, *i.e.*, *φ*^*G*^(***X****_k_*) − *φ*^*G*^(***X_k_***_−1_) = Δ*φ_k_*). This property is verified by the simulation results.

However, ***X****_k_* and ***X_k_***_−1_ are unknown and ***X̅****_k_* is also unavailable at the beginning of period *k*, thus we use ***X̅_k_***_−1_ and ***X̅_k_***_−2_ to approximate ***X****_k_* and ***X_k_***_−1_. $\theta_{k}^{A}$ is then calculated according to the change of orientation angles of the ellipse represented by ***X̅****_k_*_−1_ and ***X̅****_k_*_−2_

From the above conditions, $\theta_{k}^{A}$ can be calculated by:

(39) $$\theta_{k}^{A}=\varphi^{\text{G}}({\bar{X}}_{k-1})-\varphi^{\text{G}}({\bar{X}}_{k-2}),\;k>2$$

The next task is to determine $\gamma_{k}^{A,\cdot }\cdot \gamma_{k}^{A,\cdot }$ can be determined by the ratios of the corresponding eigenvalues of ***X̅_k_***_−1_ and ***X̅_k_***_−2_. When the size of the object does not change rapidly and sharply, the following $\gamma_{k}^{A,\cdot }$ calculation method is suggested.

(40) $$\gamma_{k}^{A,1}=\gamma_{k}^{A,2}=\sqrt{\text{tr}({\bar{X}}_{k-1})/\text{tr}({\bar{X}}_{k-2})},\;k>2$$

where tr(·) is short for trace(·).

Therefore, ***A****_k_* can be adaptively determined according to Equations (36), (39) and (40).

### 3.2. Measurement Noise Model Parameter Adaption

Let $\sum_{k}^{v}$ denote the real covariance matrix of measurement noise $\nu_{k}^{r}$. According to Equation (9), $B_{k}X_{k}B_{k}^{T}$ is used to approximate $\sum_{k}^{v}$. Thus, the basic idea of ***B****_k_* adaption is adjusting ***B****_k_* to meet $B_{k}X_{k}B_{k}^{T}$ = $\sum_{k}^{v}$ as accurately as possible. Given that ***X****_k_* and $\sum_{k}^{v}$ are unknown and ***X̅****_k_* is also unavailable at the beginning of period *k*, ***B****_k_* is calculated according to the difference between ***X̅****_k_*_−1_(*k* > 1) and $\sum_{k}^{*}$ , where $\sum_{k}^{*}$ is an approximation of $\sum_{k}^{v}$. Let ***Ψ****_k_* denote the noise covariance matrix of measurement sensor, which is usually known as prior information. Then $\sum_{k}^{*}$ is calculated by [3]:

(41) $$\Sigma_{k}^{*}=\eta {\bar{X}}_{k|k-1}+\Psi_{k}$$

where *η* is a scaling coefficient, ***X̅_k_***_|_*_k_*_−1_ is utilized to approximate ***X****_k_*, and ${\bar{X}}_{k|k-1}=\frac{X_{k|k-1}}{\left(v_{k|k-1}-2d-2\right)}$according to Equation (14).

Similar to Equation (36) ***B****_k_* is calculated by:

(42) $$B_{k}=R(\theta_{k}^{B})\text{diag}(\gamma_{k}^{B,1},\gamma_{k}^{B,2})$$

$\theta_{k}^{B}$ and $\gamma_{k}^{B,\cdot }$ in Equation (42) can be approximately calculated in a manner similar to $\theta_{k}^{A}$and $\gamma_{k}^{A,\cdot }$ calculation in Equations (39) and (40):

(43) $$\theta_{k}^{B}=\varphi^{\text{G}}(\sum_{k}^{*})-\varphi^{\text{G}}({\bar{X}}_{k-1}),\;k>1$$

(44) $$\gamma_{k}^{B,1}=\gamma_{k}^{B,2}=\sqrt{\text{tr}(\Sigma_{k}^{*})/\text{tr}({\bar{X}}_{k-1})},\;k>1$$

### 3.3. Model Parameter Adaption When d = 3

For 3D EOT (*d* = 3), ***X****_k_* can be transformed to a diagonal matrix by up to three “Givens rotations”, which means that at most three angles are required to determine a 3 × 3 SPD matrix [17]. Thus, the following equation is introduced to calculate ***A****_k_*:

(45) $${\text{A}}_{k}={\text{R}}_{3}(\theta_{k}^{A,3}){\text{R}}_{2}(\theta_{k}^{A,2}){\text{R}}_{1}(\theta_{k}^{A,1})\text{diag}(\gamma_{k}^{A,1},\gamma_{k}^{A,2},\gamma_{k}^{A,3})$$

where $\text{R}.(\theta_{k}^{A,\cdot })$ is the 3 × 3 rotation matrix.

The calculation of $\theta_{k}^{A,\cdot }$ and $\gamma_{k}^{A,\cdot }$ in Equation (45) is similar to that in 2D EOT. However, it should be noted that the three rotation matrices in Equation (45) must be maintained in the same order during the whole EOT process. In addition, when utilizing Lemma 1 to decompose ***X****_k_*, the order of rotation matrices should be the same as that of Equation (45). The adaptive approach of ***B****_k_* when *d* = 3 is similar to that of ***A****_k_*.

## 4. Multi-Model Algorithm

The proposed ARMF approach is integrated into a multi-model algorithm framework [18] (e.g., the widely used interacting multi-model (IMM) algorithm [19]) to further improve the estimation performance.

### 4.1. Moment Matching

Moment matching is applied in step initialization and fusion of IMM. Unlike the standard IMM algorithm [18], moment matching in ARMF-MM algorithm involves both kinematic state ***x****_k_* and physical extension ***X****_k_*; this condition makes the moment matching method more complex.

Step fusion is regarded as an example in this section to introduce moment matching method. In the first place, the substitution of Equation (27) into Equation (26) yields:

(46) $$p[{\text{x}}_{k},{\text{X}}_{k}|{\text{Z}}^{k}]\propto N({\text{x}}_{k};{\text{x}}_{k|k},{\text{P}}_{k|k})IW({\text{X}}_{k};v_{k|k},\alpha_{k|k}{\bar{\text{X}}}_{k})$$

Moment matching in step fusion requires that the following approximation be solved:

(47) $$\begin{array}{l} \sum_{j=1}^{n}\mu_{k}^{j}\times N({\text{x}}_{k};{\text{x}}_{k|k}^{j},{\text{P}}_{k|k}^{j})IW({\text{X}}_{k}^{j};v_{k|k}^{j},{\text{X}}_{k|k}^{j})\\ =\sum_{j=1}^{n}\mu_{k}^{j}\times N({\text{x}}_{k};{\text{x}}_{k|k}^{j},{\text{P}}_{k|k}^{j})IW({\text{X}}_{k}^{j};v_{k|k}^{j},\alpha_{k|k}^{j}{\bar{\text{X}}}_{k}^{j})\\ \approx N({\text{x}}_{k};{\text{x}}_{k|k},{\text{P}}_{k|k})IW({\text{X}}_{k};v_{k|k},{\text{X}}_{k|k})\\ =N({\text{x}}_{k};{\text{x}}_{k|k},P_{k|k})IW({\text{X}}_{k};v_{k|k},\alpha_{k|k}{\bar{\text{X}}}_{k}) \end{array}$$

where *j* = 1,2….,*n* denotes model *j* and *n* is the number of models.

The moment matching method for kinematic state ***x****_k_* is similar to standard IMM:

(48) $$\begin{array}{l} {\text{x}}_{k|k}=\sum_{j=1}^{n}\mu_{k}^{j}{\text{x}}_{k|k}^{j}\\ {\text{P}}_{k|k}=\sum_{j=1}^{n}\mu_{k}^{j}({\text{P}}_{k|k}^{j}+({\text{x}}_{k|k}^{j}-{\text{x}}_{k|k}){\left({\text{x}}_{k|k}^{j}-{\text{x}}_{k|k}\right)}^{\text{T}}) \end{array}$$

where $\mu_{k}^{j}≜p[m_{k}^{j}|{\text{Z}}^{k}]$ and $m_{k}^{j}$ indicates that model *j* is in effect at scan *k*.

The problem of moment matching method for physical extension is that the method considers ***X****_k|k_* and ***ν****_k|k_* in Equation (25) simultaneously. The mean square error *ê_k_* of extension estimation is first defined as follows [3]:

(49) $$\begin{array}{ll} {\hat{e}}_{k} & ≜\text{tr}(E[{\left({\bar{\text{X}}}_{k}-{\text{X}}_{k}\right)}^{2}|{\text{Z}}^{k}])=\text{tr}(E[({\text{X}}_{k}^{2}|{\text{Z}}^{k}]-{\bar{\text{X}}}_{k}^{2})\\ & =\text{tr}\left(\frac{\alpha_{k|k}\text{tr}({\bar{\text{X}}}_{k}){\bar{\text{X}}}_{k}+(\alpha_{k|k}+2){\bar{\text{X}}}_{k}^{2}}{\left(\alpha_{k|k}+1)(\alpha_{k|k}-2\right)}\right)\\ & =\frac{\alpha_{k|k}{\left(\text{tr}({\bar{\text{X}}}_{k})\right)}^{2}+(\alpha_{k|k}+2)\text{tr}({\bar{\text{X}}}_{k}^{2})}{\left(\alpha_{k|k}+1)(\alpha_{k|k}-2\right)} \end{array}$$

Expand Equation (49), and the following equation is derived [3,20]:

(50) $$v_{k|k}=\frac{{\hat{p}}_{k}+\sqrt{{\hat{p}}_{k}^{2}+4{\hat{q}}_{k}{\hat{e}}_{k}}}{2{\hat{e}}_{k}}+2d+2$$

where:

(51) $$\begin{array}{l} {\hat{p}}_{k}={\hat{e}}_{k}+{\left(\text{tr}\left({\bar{\text{X}}}_{k}\right)\right)}^{2}+\text{tr}\left({\bar{\text{X}}}_{k}^{2}\right)\\ {\hat{q}}_{k}=2\left[{\hat{e}}_{k}+\text{tr}\left({\bar{\text{X}}}_{k}^{2}\right)\right] \end{array}$$

Detailed derivation and explanation can be found in [3,20]. Then the moment matching method for physical extension is as follows:

(52) $$\begin{array}{l} {\bar{\text{X}}}_{k}=\sum_{j=1}^{n}\mu_{k}^{j}{\bar{\text{X}}}_{k}^{j}\\ {\hat{e}}_{k}=\sum_{j=1}^{n}\mu_{k}^{j}({\hat{e}}_{k}^{j}+\text{tr}({\left({\bar{\text{X}}}_{k}^{j}-{\bar{\text{X}}}_{k}\right)}^{2})) \end{array}$$

After ***X̅****_k_* and *ê_k_* are determined, ***ν***_*k|k*_ is calculated based on Equations (50) and (51). Thus, ***X***_*k|k*_ is obtained as follows:

(53) $${\text{X}}_{k|k}=\alpha_{k|k}{\bar{\text{X}}}_{k}=(v_{k|k}-2d-2){\bar{\text{X}}}_{k}$$

### 4.2. ARMF-Based Multi-Model Algorithm

The four steps of ARMF-IMM algorithm are summarized below:

(1) Reinitialization
  Let *π*^*i,j*^ denote the probability of transiting from model *i* to model *j*. $\pi_{k-1}^{i|j}$ is the probability that model *i* is in effect at scan *k* − 1 given $m_{k}^{j}$ and ***Z***^*k*−1^:
  (54) $$\begin{array}{ll} \pi_{k-1}^{i|j} & =p[m_{k-1}^{i}|m_{k}^{j},{\text{Z}}^{k-1}]\\ & =\frac{p[m_{k}^{j}|m_{k-1}^{i},{\text{Z}}^{k-1}]p[m_{k-1}^{i}|{\text{Z}}^{k-1}]}{p[m_{k}^{j}|{\text{Z}}^{k-1}]}\\ & =\frac{1}{c}\pi^{i,j}\mu_{k-1}^{i} \end{array}$$
  Reinitialization of each model is achieved by replacing corresponding probability variables in Equations (48)–(53):
  (55) $$\begin{array}{l} {\text{x}}_{k-1|k-1}^{0j}=\sum_{i=1}^{n}\pi_{k-1}^{i|j}{\text{x}}_{k-1|k-1}^{i}\\ {\text{P}}_{k-1|k-1}^{0j}=\sum_{i=1}^{n}\pi_{k-1}^{i|j}\left({\text{P}}_{k-1|k-1}^{i}+({\text{x}}_{k-1|k-1}^{i}-{\text{x}}_{k-1|k-1}^{0j}){\left(•\right)}^{T}\right)\\ {\bar{\text{X}}}_{k-1}^{0j}=\sum_{i=1}^{n}\pi_{k-1}^{i|j}{\bar{\text{X}}}_{k-1}^{i}=\sum_{i=1}^{n}\left(\frac{\pi_{k-1}^{i|j}{\text{X}}_{k-1|k-1}^{i}}{v_{k-1|k-1}^{i}-2d-2}\right)\\ {\hat{e}}_{k-1}^{0j}=\sum_{i=1}^{n}\pi_{k-1}^{i|j}\left({\hat{e}}_{k-1}^{i}+\text{tr}\left[{\left({\bar{\text{X}}}_{k-1}^{i}-{\bar{\text{X}}}_{k-1}^{0j}\right)}^{2}\right]\right)\\ {\text{X}}_{k-1|k-1}^{0j}=(v_{k-1|k-1}^{0j}-2d-2){\bar{\text{X}}}_{k-1}^{0j} \end{array}$$
(2) Model Parameter Adaption and Filtering
  For model *j*, *j* = 1,2….,*n*, calculate ${\text{A}}_{k}^{j}$ according to Equations (36), (39) and (40). Then the RMF approach as summarized in Section 2 is performed and ${\text{B}}_{k}^{j}$ is calculated according to Equations (42)–(44) for model *j*.
(3) Probability Update
  According to Equation (54), probability $\mu_{k}^{j}$ is calculated by:
  (56) $$\begin{array}{ll} \mu_{k}^{j} & =p[m_{k}^{j}|{\text{Z}}^{k}]=\frac{1}{c}p[{\text{Z}}_{k}|m_{k}^{j},{\text{Z}}^{k-1}]p[m_{k}^{j}|{\text{Z}}^{k-1}]\\ & ≜\frac{1}{c}\Lambda_{k}^{j}p[m_{k}^{j}|{\text{Z}}^{k-1}]\\ & \frac{1}{c}\Lambda_{k}^{j}(\sum_{i=1}^{n}\pi^{i,j}\mu_{k-1}^{i}) \end{array}$$
  Likelihood $\Lambda_{k}^{j}$ is derived as:
  (57) $$\begin{array}{ll} \Lambda_{k}^{j} & ≜p[{\text{Z}}_{k}|m_{k}^{j},{\text{Z}}^{k-1}]\\ & =\int \int p[{\text{Z}}_{k}|m_{k}^{j},{\text{x}}_{k},{\text{X}}_{k}]p[{\text{x}}_{k},{\text{X}}_{k}|m_{k}^{j},{\text{Z}}^{k-1}]d{\text{x}}_{k}d{\text{X}}_{k} \end{array}$$
  where $p\left[x_{k},{\text{X}}_{k}|m_{k}^{j},{\text{Z}}^{k-1}\right]$ is calculated by Equation (17) and $p\left[{\text{Z}}^{k}|m_{k}^{j},x_{k}{\text{X}}_{k}\right]$ by Equation (18).
  The substitution of Equations (17) and (18) into Equation (57) approximately yields:
  (58) $$\begin{array}{l} \begin{array}{ll} \Lambda_{k}^{j} & =N({\bar{z}}_{k};{\text{H}}_{k}{\text{x}}_{k|k-1}^{j},{\text{S}}_{k|k-1}^{j})\times \frac{\Gamma_{d}\left[(n_{k}+v_{k|k-1}^{j}-d-2)/2\right]}{\Gamma_{d}\left[(v_{k|k-1}^{j}-d-1)/2\right]}\\ & \times {\left|{\text{B}}_{k}^{j}{\text{X}}_{k|k-1}^{j}{\left({\text{B}}_{k}^{j}\right)}^{\text{T}}\right|}^{\frac{v_{k|k-1}^{j}-d-1}{2}}\times {\left|{\text{B}}_{k}^{j}{\text{X}}_{k|k-1}^{j}{\left({\text{B}}_{k}^{j}\right)}^{T}+{\bar{\text{Z}}}_{k}\right|}^{\frac{-(n_{k}+v_{k|k-1}^{j}-d-2)}{2}} \end{array} \end{array}$$
  where ${\text{X}}_{k|k-1}^{j}$ is used to approximate ${\text{X}}_{k}^{j}$ in ${\text{S}}_{k|k-1}^{j}$, and Γ_d_[**·**] is the multivariate gamma function. The derivation of $\Lambda_{k}^{j}$ is quite similar to that of Equations (11) and (13). When *n_k_* = 1, $\Lambda_{k}^{j}=N({\bar{\text{z}}}_{k};{\text{H}}_{k}{\text{x}}_{k|k-1}^{j},{\text{S}}_{k|k-1}^{j})$.
(4) Fusion
  The moment matching formulas of fusion are provided by Equations (48)–(53). The final result of extension estimation is ***X̄****_k_* defined in Equation (27).

## 5. Simulation Results and Discussions

Two typical EOT scenarios are simulated in this section to compare the proposed ARMF approach and other RMF approaches.

### 5.1. Scenario 1

**Figure 2.**
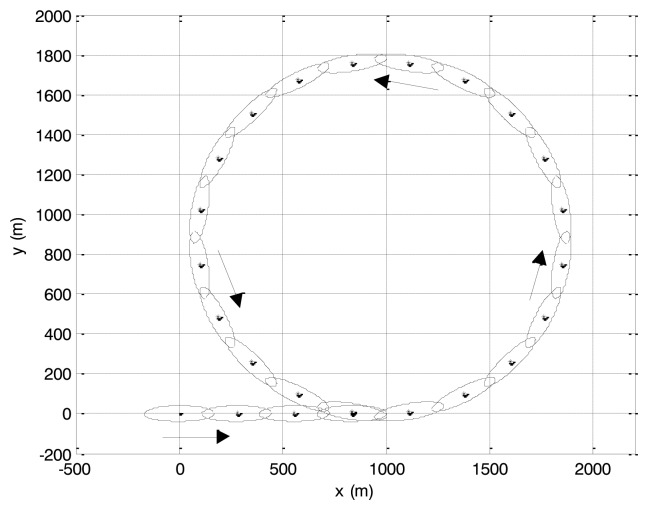
Target trajectory of simulation scenario 1.

The route of an aircraft carrier, which is approximately 300 m long and 80 m wide, is shown in Figure 2. The route starts from origin (0, 0)^T^, moves along the trajectory, and then maintains a uniform circular motion on the 2D plane. Constant velocity is set to 27 knots (approximately 50 km/h). The aircraft carrier's extension is characterized by an ellipse with radiuses 170 m and 40 m in RMF approach. Scan period *T* is set to 10 s. The measurement scattering centers are assumed to be distributed uniformly over the physical extension (*i.e.*, the ellipse) [3], and the number of measurements in every scan obeys Poisson distribution with mean 20. The sensor measurement noise follows a zero-mean Gaussian distribution with covariance matrix *Ψ_k_* = diag([50^2^,20^2^]) m^2^.

The single-model ARMF is compared with Koch's approach [1] and Feldmann's approach [3] through *N*_M_ = 500 Monte Carlo runs.

(1) *Koch's approach*: the temporal decay constant τ is set to 8*T*;
(2) *Feldmann's approach*: 0.25***X****_k_* + ***Ψ****_k_* is utilized as the measurement noise covariance matrix; and
(3) *ARMF: η* in Equation (42) is set to 0.25 via moment matching [3].

**Figure 3.**
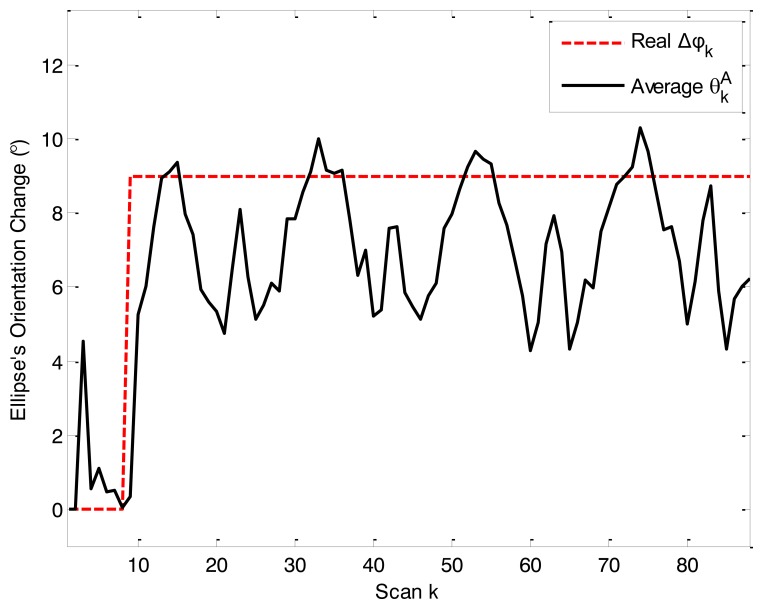
Average $\theta_{k}^{A}$ in simulation scenario 1.

Average $\theta_{k}^{A}$ , which describes the change in the ellipse's orientation angle, is shown in Figure 3. The curve shows that the model parameter adaptive approach is effective and that ARMF is able to adjust $\theta_{k}^{A}$ according to the extension's changes.

The root mean square error (RMSE) of physical extension ***X_k_*** is defined by [3]:

(59) $$\text{RMS}E_{X}=\sqrt{\frac{1}{N_{M}}\sum_{l=1}^{N_{M}}\text{tr}\left[{\left({\bar{\text{X}}}_{k}^{l}-{\text{X}}_{k}\right)}^{2}\right]}$$

where ${\bar{X}}_{k}^{l}$ stands for the expectation of physical extension in *l*th Monte Carlo run.

**Figure 4.**
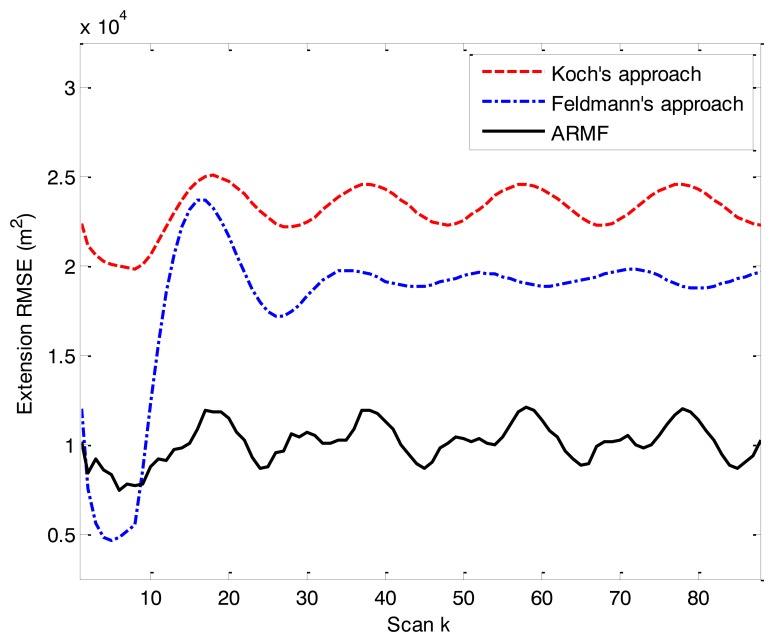
RMSE of physical extension in simulation scenario 1.

The RMSE of physical extension of the three RMF approaches is shown in Figure 4. When the object makes a turn (uniform circular motion after scan 10), the extension RMSE of ARMF becomes lower than that of the other two approaches.

### 5.2. Scenario 2

**Figure 5.**
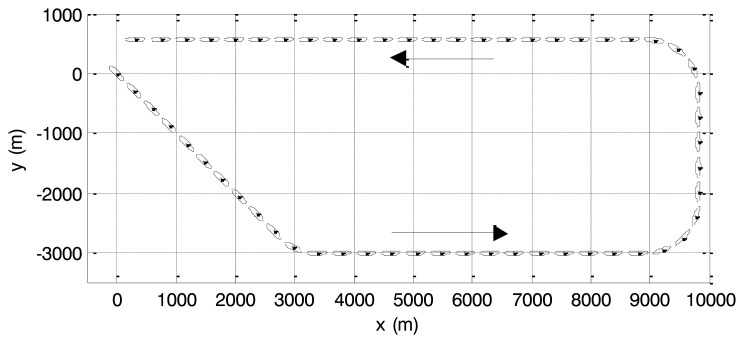
Target trajectory of simulation scenario 2.

The extended object scenario in [3] is simulated below. The aircraft carrier whose size is the same as the one in scenario 1 starts from the origin and moves along the trajectory on the 2D plane as shown in Figure 5. Three turns are implemented.

The following three approaches are compared through *N*_M_ = 500 Monte Carlo runs.

(1) *Koch's approach*: parameters are the same as those in scenario 1;
(2) *Feldmann's MM approach*: parameters are designed similar to those in [3]; and
(3) *ARMF-MM*: three models are used. Model 1 with low kinematic process noise and low extension agility (small *Q_k_*, large *δ_k_*), model 2 with high kinematic process noise and high extension agility (large *Q_k_*, small *δ_k_*), and model 3 with moderate kinematic process noise and high extension agility (moderate *Q_k_*, small *δ_k_*) are utilized. *η*=0.25 for all three models.

**Figure 6.**
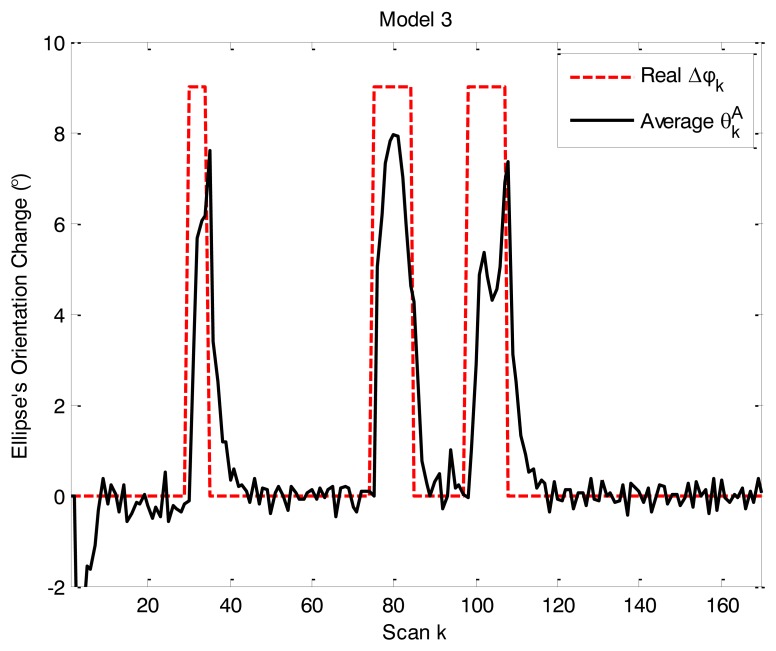
Average $\theta_{k}^{A}$ of model 3 in simulation scenario 2.

The simulation results reconfirmed the validity of the model parameter adaptive approach. ARMF-MM identified the angle changes correctly when the object maneuvers as shown in Figure 6.

**Figure 7.**
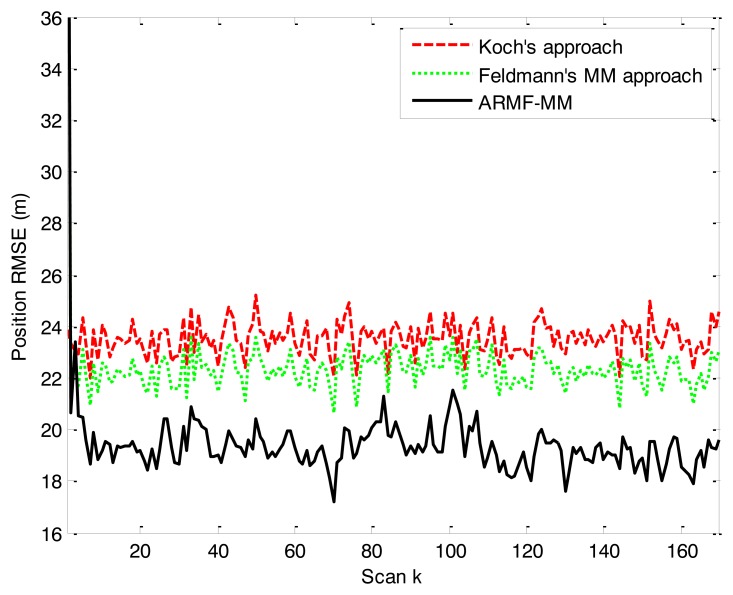
RMSE of position in simulation scenario 2.

**Figure 8.**
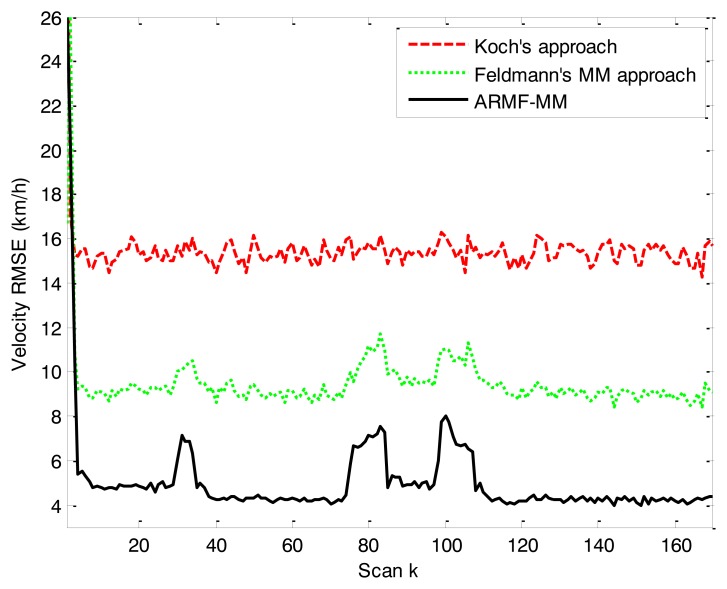
RMSE of velocity in simulation scenario 2.

**Figure 9.**
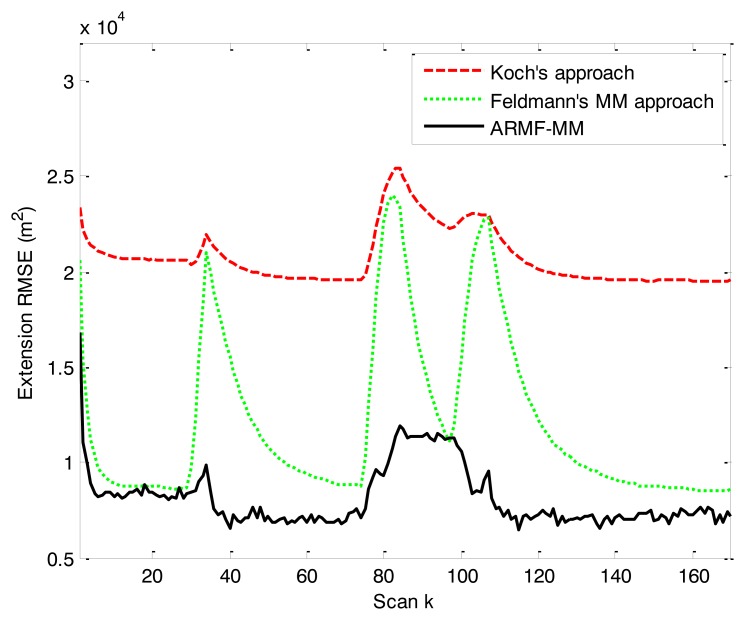
RMSE of physical extension in simulation scenario 2.

The position RMSEs of three RMFs are close to one another. The error curve is shown in Figure 7. The position RMSE of ARMF-MM is lower than that of the others. The RMSEs of velocity and physical extension are shown in Figures 8 and 9. ARMF-MM is able to maintain a low level of velocity RMSE in the entire process and performs better than the others in physical extension estimation. The physical extension RMSE of ARMF-MM is significantly reduced when the target maneuvers.

## 6. Conclusions

Bayesian EOT using a random matrix is a very effective and simple approach to incorporate physical extension in the OT framework. The Bayesian RMF approach was reviewed first in this paper, and certain improvements were made. Afterward, model parameter adaptive approaches were derived for both extension dynamic evolution and measurement noise based on the analysis of SPD matrix decomposition. The proposed adaptive RMF can be integrated into an IMM framework to further improve estimation performance. The validity of ARMF and ARMF-MM was verified by the simulation results. ARMF is able to change model parameters adaptively when the extended object maneuvers, thereby resulting in low physical extension estimation error.
